# Supplementary material for: Pinocembrin Ameliorates Skin Fibrosis via Inhibiting TGF-β1 Signaling Pathway
Source: Biomolecules. 2021 Aug 19;11(8):1240. doi: 10.3390/biom11081240 (PMC8393190; doi:10.3390/biom11081240)
Supplement: Supplementary file 1 [file biomolecules-11-01240-s001.zip › biomolecules-1239663-supplementary.pdf]

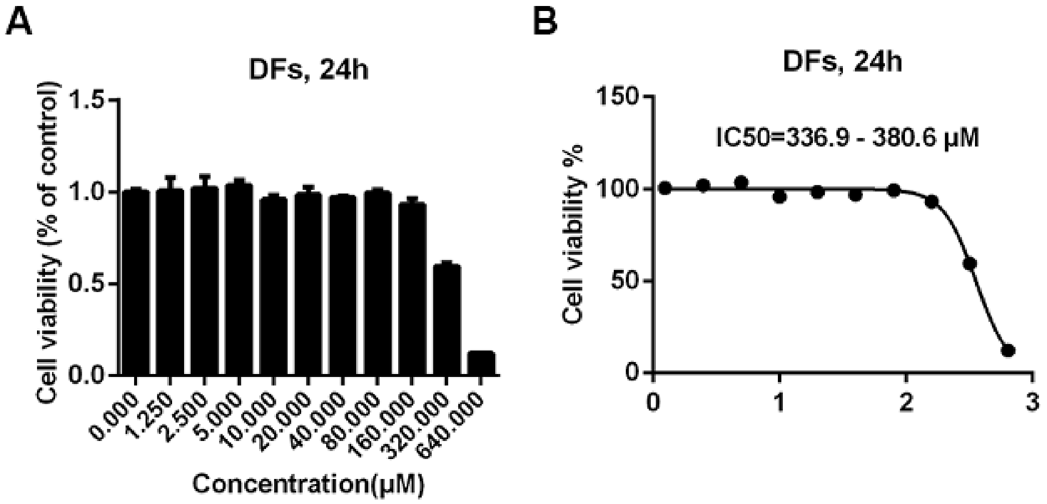

Figure S1. MTT assays of mouse primary dermal fibroblasts treated with pinocembrin. Cells were exposed to the indicated doses of pinocembrin (0 to 640 μM) for 24 h, IC50 = 336.9-380.3 μM, (n = 3 per group).

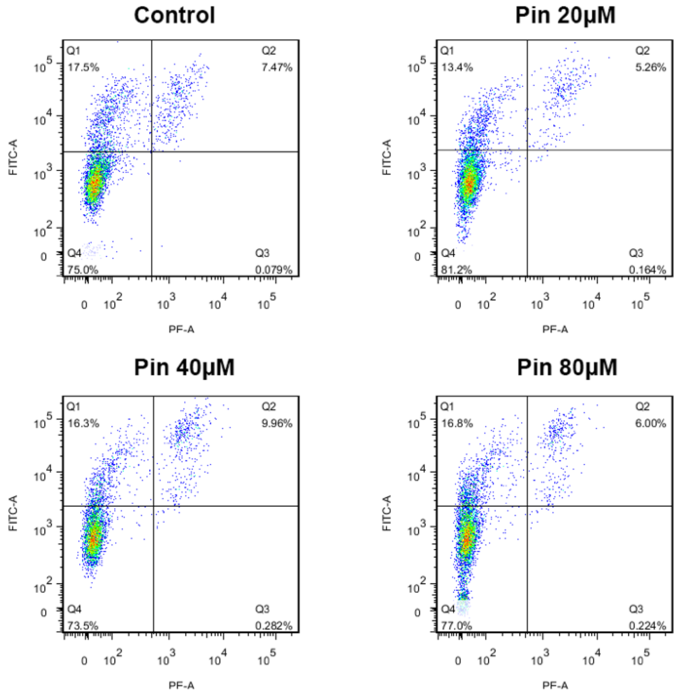

Figure S2. Flow cytometry assay of mouse primary dermal fibroblasts treated with pinocembrin. The mouse primary dermal fibroblasts were treated with indicated doses of pinocembrin (20 μM, 40 μM, 80 μM) for 48h and Annexin V/PI staining was subsequently performed to estimate apoptosis and necrosis by flow cytometry.

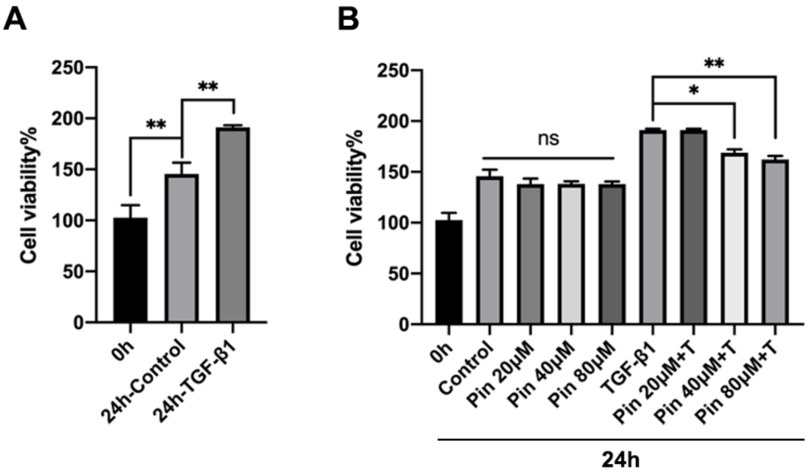

Figure S3. Pinocembrin inhibits TGF-β1-induced vitality increase of mouse primary dermal fibroblasts. (A) Cells were treated with or without TGF-β1 (5 ng/ml) for 24 h and the cell viability was detected by MTT assay. (B) Cells were exposed to the indicated doses of pinocembrin (20 μM, 40 μM, 80 μM) and co-treated with or without TGF-β1 (5 ng/ml) for 24 h and the cell viability was detected by MTT assay. (n = 3 per group).

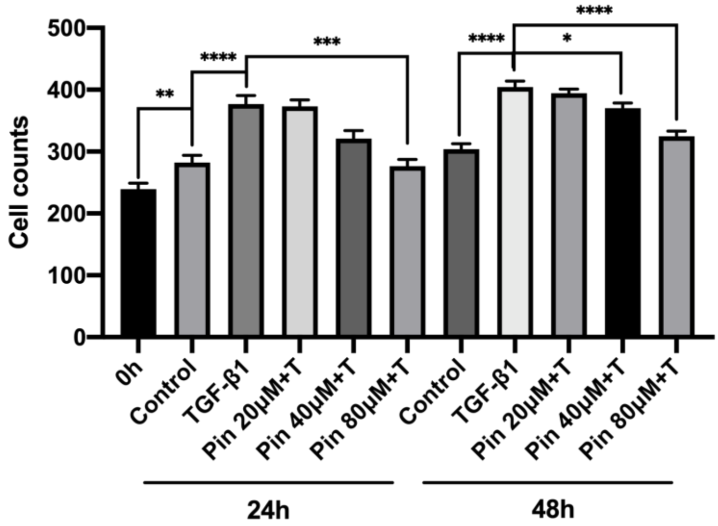

Figure S4. Pinocembrin inhibits TGF-induced proliferation of mouse primary dermal fibroblasts. Cells were treated with indicated doses of pinocembrin (20 μM, 40 μM, 80 μM) and co-treated with or without TGF-β1 (5 ng/ml) for 24 h or 48 h and the cell counts were obtained based on the microscope picture. (n = 3 per group).

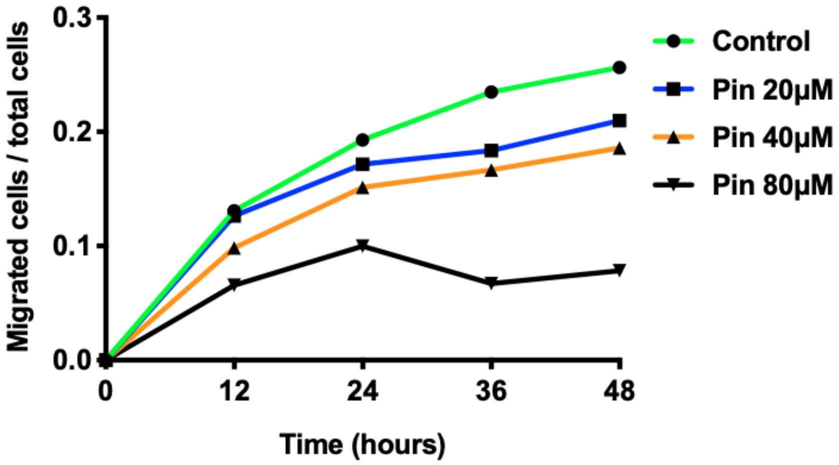

Figure S5. Pinocembrin suppresses the migration of keloid fibroblasts. The wound healing assay was used to analysis the migration of KFs treated with pinocembrin (0, 20, 40, 80 μM). The ratio of migrated cells to total cells were calculated in 12, 24, 36, and 48 h post scratching.

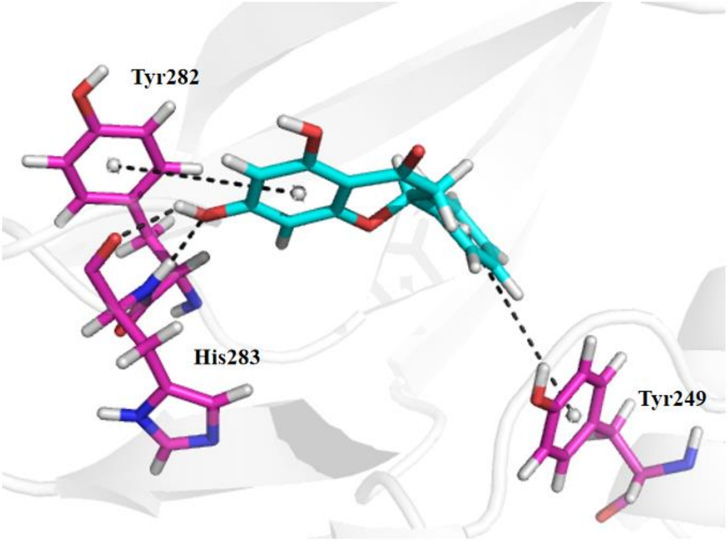

Figure S6. Molecular docking assay of Pinocembrin and ALK5. The 5'-hydroxyl group of Pinocembrin forms two hydrogen bonds with the backbone of His283 and the two aromatic rings of Pinocembrin interact with Tyr249 and Tyr282 through π-π stacking.
